# Supplementary material for: Tissueformer: extending single-cell foundation models to predict population-level phenotypes
Source: BMC Bioinformatics. 2026 Jun 4;27:172. doi: 10.1186/s12859-026-06490-4 (PMC13466306; doi:10.1186/s12859-026-06490-4)
Supplement: Supplementary file 1 [file 12859_2026_6490_MOESM1_ESM.pdf]

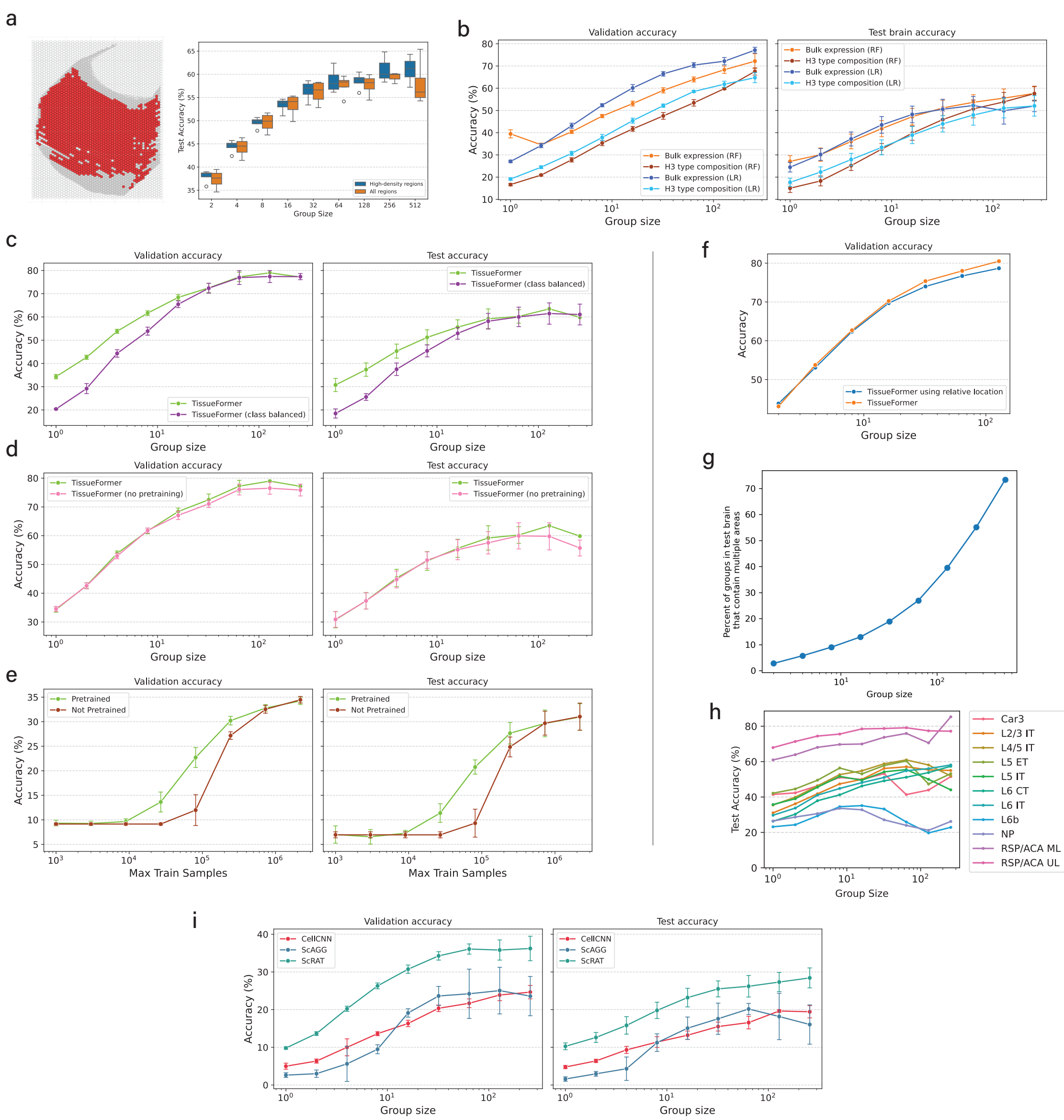

Figure 2—figure supplement 1. a) The drop in test brain accuracy relative to validation accuracy is only partially due to differences in sampling density. We selected hexes in the test brain which had high density in the 3 training brains (example at left), specifically, hexes with at least as many cells as the group size. (right) Test accuracy on data in those hexes was marginally higher than for all points, yet still much below validation accuracy. **b)** Performance of random forests (RF) compared against logistic regression (LR) given identical inputs. **c)** Performance of a class-balanced model which controls for some areas having more cells in training brains with a modified objective that discounts samples from frequent areas. **d)** Effect of initializing TissueFormer with a pretrained Murine Geneformer as single-cell module vs. randomly initialized. **e)** Varying the number of train samples, comparing random initalizations to pretrained initializations, here for $N = 1$ group size. $N = 1$ was chosen to reduce ambiguity and ensure that the number of cells seen equaled the number of groups seen. **f)** Effect of giving TissueFormer the relative spatial location (relative to the center of mass) within each group of each cell. **g)** Number of groups in the test brain that contain an area boundary as a function of the group size. **h)** The accuracy curves of models trained while only ever able to observe groups containing a single cell type. Groups were constructed by selecting a single cell and choosing the nearest N cells of the same time. The high performance of some types, such as RSP/ACA, reflects the fact that these cells are localized in just one area of cortex (retrospenial/anterior cingulate areas). Slopes of these curves are shown in Fig. 2. **i)** Validation (left) and test (right) accuracy as a function of group size for three deep learning benchmarks: CellCnn, scAGG, and ScRAT. TissueFormer (shown in **Figure 2**f-g) outperforms all three methods.

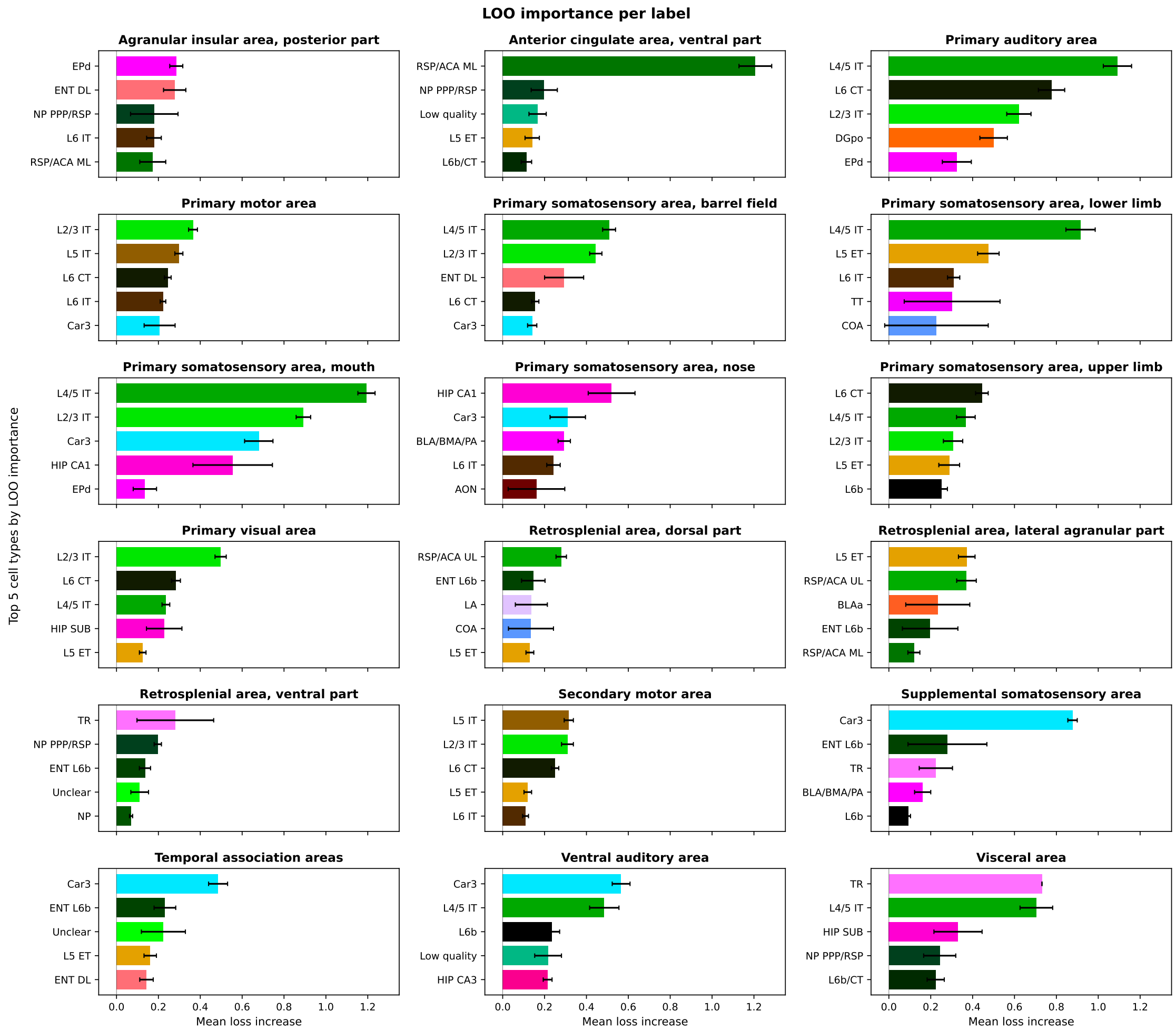

Figure 2—figure supplement 2. Leave-one-cell-type-out (LOO) analysis for the brain annotation task. Each panel shows the top cell types by LOO importance for a given predicted brain area. Importance is measured as the mean increase in cross-entropy loss when all cells of that type are removed from a group. L4/5 IT cells are consistently important across somatosensory areas, while L2/3 IT cells are most important for visual areas. Error bars represent standard error of the mean across groups.

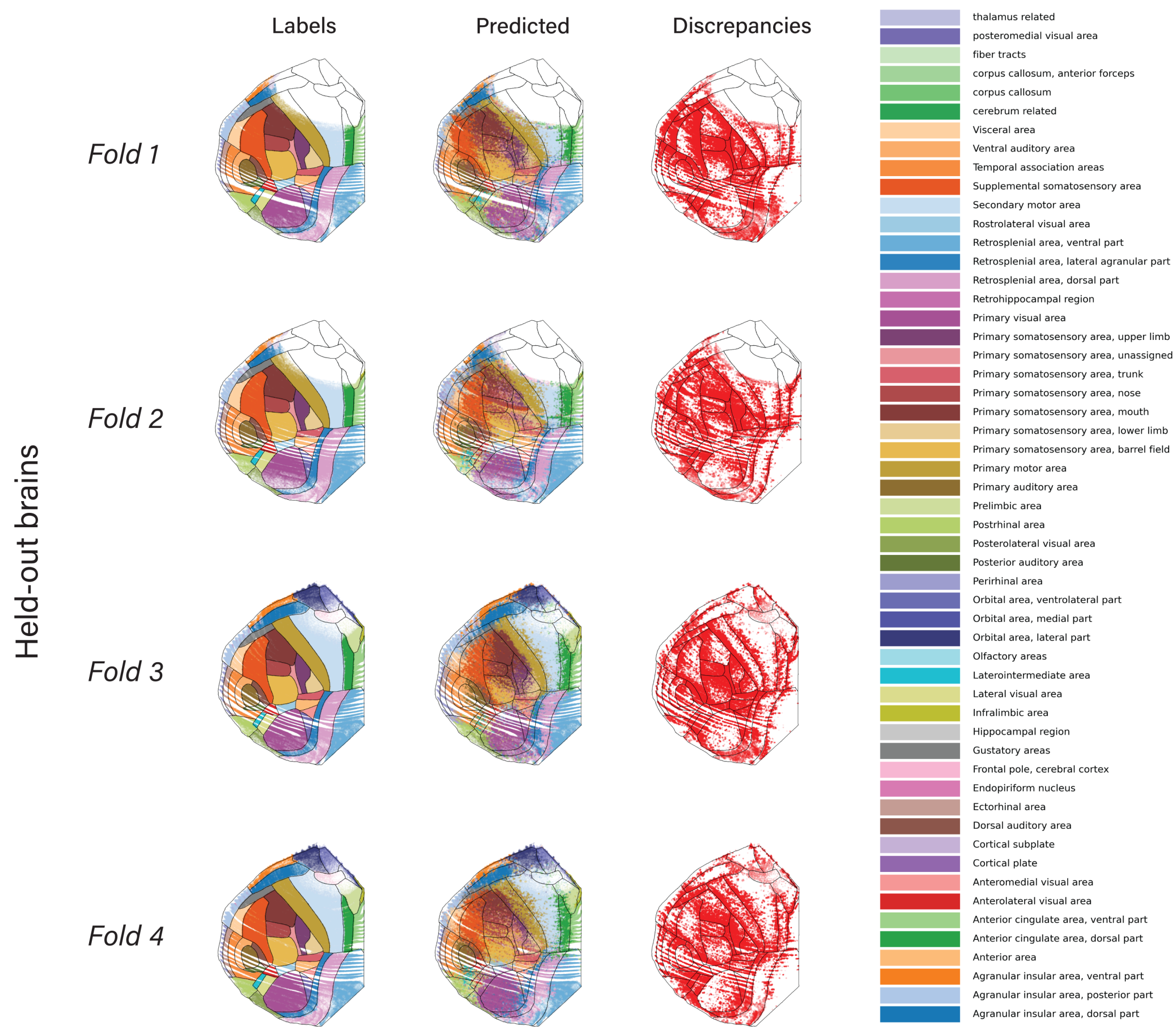

Figure 3—figure supplement 1. Across all 4 brains, we show the CCF labels on single cells (left), the predicted labels (middle) and the location of mismatches between the two. We call these discrepancies rather than errors as it is not clear which is closer to ground truth. Notable systematic differences include a gross shift of the Primary Visual Area (magenta), and the somatosensory-motor boundary. In both cases, a shift appears in the discrepancy map as a large amount of discrepancies on one side of an area but very few on the other side, and a large discontinuity at the CCF boundary.

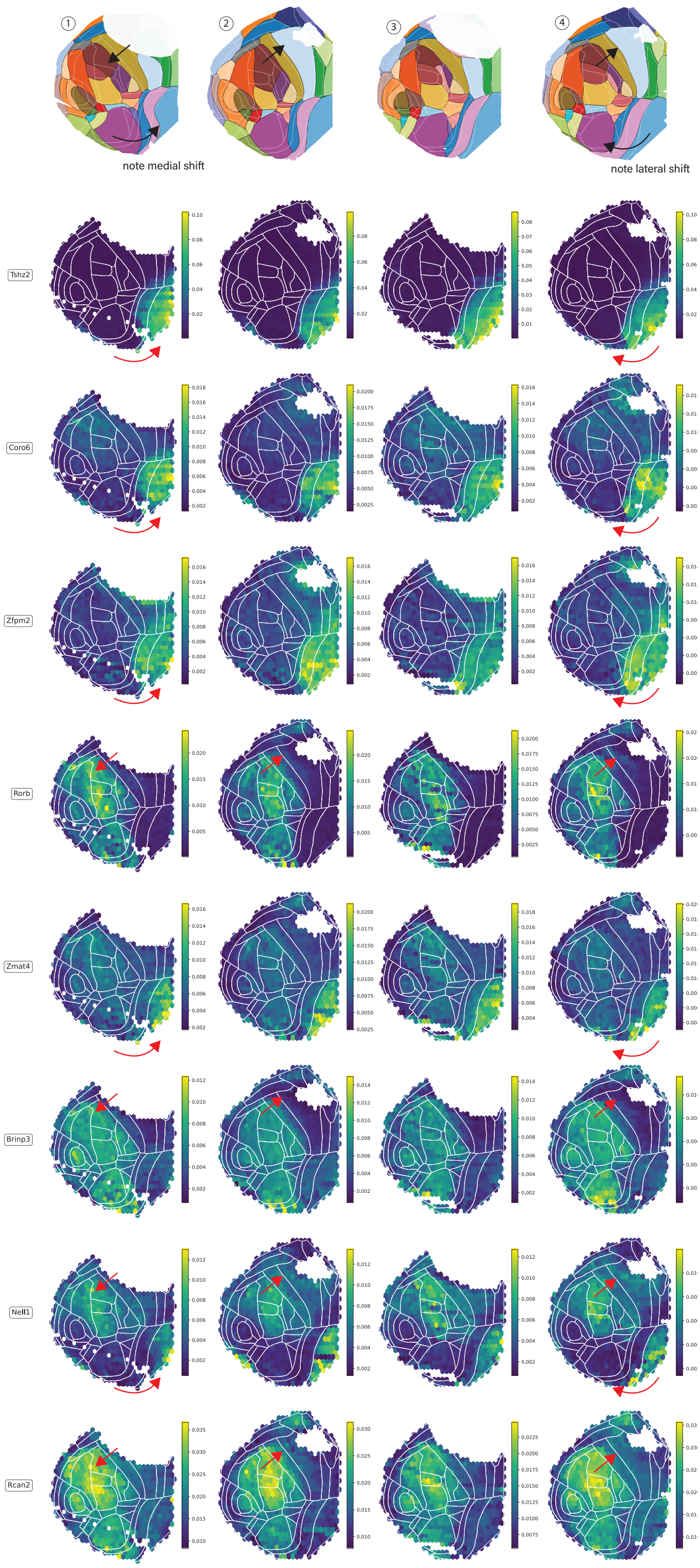

Figure 3—figure supplement 2. Normalized spatial density of select genes in each animal. Arrows highlight the animal-specific shifts of regional anatomy relative to the CCF (white boundaries), and how these are consistent between genes and with the predictions of Tissueformer (top row, reproduced from **Figure 3**. The top three rows (Tshz2, Coro6, and Zfmp2) recapitulate the medial shift in the boundary to the retrospenial areas in Brain 1 and the lateral shift in this boundary in Brain 4. The bottom 5 rows recapitulate the shift in the somatosensory/motor boundary, the same as analyzed in cell type distributions in Figure 3. To construct normalized gene densities, we first normalized mRNA transcription levels within each cell. We then plot the average normalized transcription within each hex. Normalization was necessary to reduce confounds of unequal spatial sampling.
